# Impacts of Cancer on Platelet Production, Activation and Education and Mechanisms of Cancer-Associated Thrombosis

**DOI:** 10.3390/cancers10110441

**Published:** 2018-11-14

**Authors:** Léa Plantureux, Diane Mège, Lydie Crescence, Françoise Dignat-George, Christophe Dubois, Laurence Panicot-Dubois

**Affiliations:** 1 Aix Marseille Univ, INSERM 1263, INRA 1260, C2VN, Faculty of pharmacy, 27 Boulevard Jean Moulin, 13385 Marseille, France; l.plantureux@gmail.com (L.P.); dr.dianemege@gmail.com (D.M.); lydie.crescence@univ-amu.fr (L.C.); francoise.dignat-george@univ-amu.fr (F.D.-G.); christophe.dubois@univ-amu.fr (C.D.); 2 Department of Digestive Surgery, Timone University Hospital, 13385 Marseille, France

**Keywords:** platelets, cancer cells, tumor cell induced platelet aggregation (TCIPA), tumor educated platelets (TEP), cancer-associated thrombosis

## Abstract

Platelets are small anucleate cells that are traditionally described as the major effectors of hemostasis and thrombosis. However, increasing evidence indicates that platelets play several roles in the progression of malignancies and in cancer-associated thrombosis. A notable cross-communication exists between platelets and cancer cells. On one hand, cancer can “educate” platelets, influencing their RNA profiles, the numbers of circulating platelets and their activation states. On the other hand, tumor-educated platelets contain a plethora of active biomolecules, including platelet-specific and circulating ingested biomolecules, that are released upon platelet activation and participate in the progression of malignancy. The numerous mechanisms by which the primary tumor induces the production, activation and aggregation of platelets (also known as tumor cell induced platelet aggregation, or TCIPA) are directly related to the pro-thrombotic state of cancer patients. Moreover, the activation of platelets is critical for tumor growth and successful metastatic outbreak. The development or use of existing drugs targeting the activation of platelets, adhesive proteins responsible for cancer cell-platelet interactions and platelet agonists should be used to reduce cancer-associated thrombosis and tumor progression.

## 1. Introduction

Platelets are small (2–4 µm) anuclueate hematopoietic cells released by bone marrow megakaryocytes into the bloodstream. In healthy humans, the concentration of circulating platelets is approximately 150 to 350 × 10^9^/L. For a long time, platelets were described as the major effectors of hemostasis and thrombosis. The hemostatic functions of platelets were first described in 1873 by Osler, who showed the presence of “blood plaques” in white thrombi.

The platelet membrane is composed of phospholipids and many receptors and glycoproteins, which enable their quick adhesion, activation and aggregation that is essential for their hemostatic function. After vascular injury, platelets rapidly interact with the vessel wall in a glycoprotein (GP)Ib-V-IX–Von Willebrand Factor (VWF)-dependent manner. This interaction is followed by a firm adhesion on the subendothelial collagen through platelet-specific collagen receptor GPIV (Glycoprotein VI) and integrin α_2_ß_1_. Platelets become activated, exhibiting first an intracellular mobilization of calcium followed by shape change and degranulation. Platelets contain three types of granules: (i) dense granules containing platelet agonists, such as ADP (Adenosine diphosphate), ATP (Adenosine triphosphate) and serotonin; (ii) alpha granules containing adhesive molecules, such as fibronectin, fibrinogen, GPIb and integrin αIIbß3, coagulation factors, growth factors and chemokines; and (iii) lysosomal granules that contain proteases and glycosidases, such as collagenase and cathepsin. 

During the process of platelet activation, platelets release their granules containing platelet agonists that lead to an amplification of the activation response through specific G-coupled receptors. Moreover, local thrombin generation increases the activation response of platelets by its proteolytic activity on protease-activated receptors (PARs) present on platelets. The release of these platelet agonists enables the recruitment, adhesion and activation of neighboring platelets. Finally, this process leads to the aggregation of platelets through the linking of α_IIb_ß3 with fibrinogen and to the formation of a hemostatic plug that avoids blood loss.

In addition to their physiological hemostatic functions, in 1865, Armand Trousseau demonstrated a close relation between thrombosis and cancer [1]. In recent years, significant clinical and experimental evidence supports the finding that platelets play several roles in the progression of malignancies and in cancer-associated thrombosis [2]. Moreover, cancer can influence the platelet count, physiology, activation state and RNA profile. The abilities of tumor cells to activate and aggregate platelets give them numerous advantages in the bloodstream. Platelets may protect circulating cancer cells against the immune system, favor pro-survival signals, induce invasive properties and transfer adhesive molecules which will interact with the endothelium participating in the early metastatic niches [3,4,5,6]. Recent studies have demonstrated that cancer can educate platelets (tumor-educated platelets), providing interesting tools for cancer diagnostics. Platelets are indeed able to sequester tumor derived biomolecules including mRNA and proteins. The activation of platelets by external signals induced specific splice variants of premRNA into platelets, providing a specific spliced mRNA signature into platelets. In this review, we will discuss the impacts of cancer on platelet physiology and phenotype and its association with the pro-thrombotic states of cancer patients.

## 2. Effects of Cancer on Platelets and Mechanisms Involved in Cancer-Associated Thrombosis

### 2.1. Thrombocytosis

In 1968, Gasic and collaborators were the first group to associate platelet counts with the number of metastases [7]. Using an experimental mouse model, this group showed a close relation between neuraminidase-induced thrombocytopenia and the reduction of metastasis of TA3 ascites tumor cells through an unexpected effect of neuraminidase. This relationship was confirmed by Kapartkin et al. [8] in 1981. These researchers demonstrated that the injection of cancer cells into the bloodstream of mice induces thrombocytopenia, and it was closely correlated to their metastatic potential. However, a portion of cancer cells failed to aggregate platelets in vitro and were unable to metastasize in vivo.

**Figure 1:**
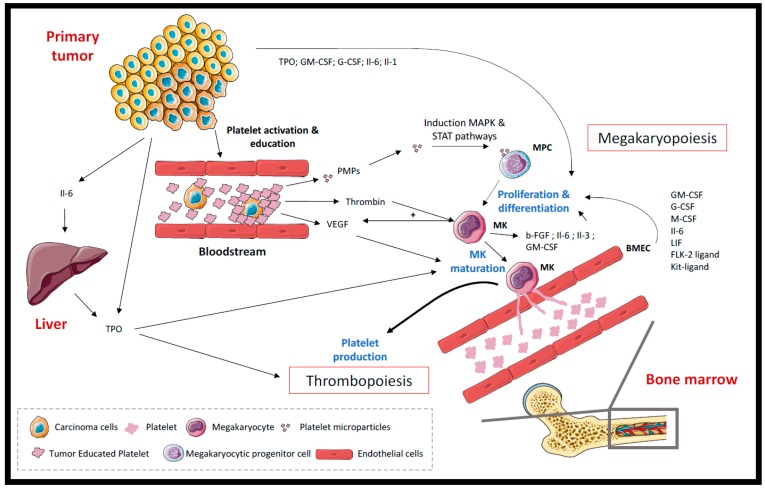
Mechanisms of cancer-associated thrombocytosis. This figure summarizes all the mechanisms involved in the production of platelets mediated by the primary tumor. BMEC: Bone Marrow Endothelial Cells. This figure was obtained using Servier medical art. http://smart.servier.com/. GM-CSF: Granulocyte-Macrophage Colony-Stimulating Factor; G-CSF: Granulocyte Colony-Stimulating Factor; M-CSF: Macrophage Colony-Stimulating Factor; Il-6: Interleukin-6; LIF: Leukemia Inhibitory Factor; FLK-2: Fetal Liver Kinase-2; Kit-ligand (Steel factor); TPO: thrombopoietin.

Alternatively, several clinical studies have demonstrated the presence of an elevated platelet count or thrombocytosis (greater than 400 × 10^9^/L) in 2.7% to 49.8% of gastrointestinal cancer patients [9], as well as in other types of solid cancer, including renal, breast, lung, colorectal and urogenital cancers [10,11,12,13,14,15]. The notion of cancer-associated thrombocytosis was first described in 1872 by Reiss et al. [16] and is now correlated with shortened survival and poor prognosis [11,17] in various types of cancer. Thrombocytosis is also positively correlated with venous thromboembolism events in cancer patients [10]. All the mechanisms of cancer-induced thrombocytosis have not been thoroughly elucidated to date. To date, based on the literature, primary tumors influenced platelet production through a direct paracrine activity on megakaryocytes and via their abilities to educate and activate platelets (Figure 1).

During cancer progression, a variety of tumor-related humoral factors and cytokines directly or indirectly influence megakaryopoiesis and thrombopoiesis. The most-described factors are granulocyte colony-stimulating factor (G-CSF), granulocyte-macrophage colony-stimulating factors (GM-CSF), basic fibroblast growth factor (b-FGF), interleukin-6 (Il-6), interleukin-1 (Il-1), and thrombopoietin (TPO). It has been shown that primary tumors can produce and secrete G-CSF and GM-CSF into the bloodstream, resulting in the stimulation of megakaryopoiesis and, subsequently, thrombopoiesis in cancer patients [18]. In addition to CSF, high levels of Il-6 and Il-1 were found in the plasma and in the culture supernatant of GM-CSF- and/or G-CSF-producing tumors obtained from patients [18]. Increasing evidence has demonstrated that Il-6 actively contributes to inflammatory and cancer-associated thrombocytosis through a thrombopoietin (TPO)-dependent mechanism [19,20]. Indeed, Stone et al. demonstrated in an ovarian cancer mouse model that the increase of hepatic thrombopoietin (TPO) production in response to tumor-derived interleukin-6 (Il-6) was responsible for elevated platelet counts [19]. The inhibition of TPO and Il-6 in tumor-bearing mice was sufficient to reverse thrombocytosis. Plasma levels of Il-6 and TPO were significantly higher in ovarian cancer patients with thrombocytosis. Moreover, platelet counts were positively correlated with plasma Il-6 and TPO levels [19]. Furthermore, TPO may be directly produced by carcinoma cells, including ovarian and hepatocellular cancer cells [21,22]. The elevation of TPO production promotes bone marrow megakaryocyte growth, differentiation and platelet production [18,19,20,21,22]. 

Within the bone marrow, a strong cooperation occurred between endothelial cells and megakaryocytes. Bone marrow endothelial cells (BMEC) released several cytokines and factors, and induced in vitro and in vivo the proliferation and differentiation of hematopoietic and megakaryocytic progenitor cells supporting megakaryopoiesis [23,24]. Megakaryocytes also secreted inflammatory cytokines, such as Il-6, Il-1, Il-3 and GM-CSF, which can in turn support megakaryopoiesis [25].

Beyond their direct abilities to secrete megakaryopoietic and thrombopoietic factors, the primary tumors can also influence platelet production through the activation of platelets. For example, platelet microparticles (PMPs), released after platelet activation, can stimulate the proliferation, survival, adhesion and chemotaxis of hematopoietic cells through the activation of various intracellular signaling cascades, such as MAPK p42/44, and STAT pathways [26]. Thus, PMPs support the intercellular cross-talk during hematopoiesis. Megakaryocytes produce essential angiogenic factors as well, such as Vascular Endothelial Growth Factor (VEGF) and basic Fibroblast Growth factor (b-FGF) [25,27]. VEGF is a specific endothelial cell mitogen and a potent angiogenic factor. Once again, the activation state of platelets within the cancer patients and the excessive thrombin generation participate in cancer-associated thrombocytosis. Indeed, the thrombin stimulation of megakaryocytes increases their secretion of VEGF three-fold [27]. Moreover, in the case of breast cancer, the education of platelets by the primary tumors enhances the ability of platelets to secrete pro-angiogenic proteins, such as VEGF, following platelet activation [28]. Within the bone marrow microenvironment, VEGF potentiates the maturation of megakaryocytes through autocrine-paracrine loops in a VEGFR-1 dependent mechanism [29]. In turn, b-FGF has been described to increase megakaryocyte colony formation in vitro [30] and to induce megakaryocyte differentiation via the regulation of megakaryocyte-stromal interactions and the increased secretion of megakaryocyte cytokines [31]. 

### 2.2. Platelets and Hemostatic System Activation

Different studies have shown that the plasma levels of soluble P-selectin (sP-sel), soluble CD40 ligand (sCD40L), thrombospondin-1 (TSP1) and ß-thromboglobulin were significantly higher in cancer patients than in healthy controls, strongly suggesting that platelets get activated during cancer progression [32,33,34,35,36]. One study also suggested that high amounts of PF-4 (Platelet Factor-4) were associated with an increased risk of venous thromboembolism (VTE) in pancreatic cancer patients [37]. Circulating platelets also expressed high levels of P-selectin at their surface in cancer patients [38]. Recently, Riedl et al. investigated the association between platelet activation biomarkers and the development of cancer-associated VTE within a cohort of 1779 patients with different types of cancer. These patients had been included in the Vienna Cancer and Thrombosis Study (CATS), a prospective and observational study on patients with newly diagnosed or progressive cancer after remission. Their results showed that sP-selectin, but not sCD40L, TSP1 nor PF-4 were associated with the risk of VTE in cancer patients [39]. Cancer cells themselves can activate platelets and the coagulation system by direct interaction in the bloodstream or indirectly via microvesicles and/or secreted factors and cytokines. The ability of cancer cells to activate platelets and the coagulation system is partly responsible for the pro-thrombotic state of cancer patients and is directly correlate with their metastatic potential in vivo and in vitro [40,41]. All of the mechanisms that induce platelets activation and aggregation are called tumor cell induced platelet aggregation (TCIPA). 

#### 2.2.1. Released Factors

• ADP

Platelet adenosine diphosphate (ADP) pools are stored in the dense granules and are released upon platelet activation. Platelets express two major ADP-specific G-coupled receptors, P2Y1 and P2Y12, which are both involved in platelet shape changes, thromboxane A_2_ (TXA_2_) release and aggregation. Various cancer cell lines, including melanoma, neuroblastoma, ovarian and breast carcinoma cells, can release ADP, which contributes to TCIPA [42,43,44,45]. Moreover, the inhibition of ADP or ADP receptor P2Y12 activity by apyrase and 2-methylthio-AMP (2-MeSAMP), respectively, reduces platelet aggregation induced by MCF-7 breast carcinoma cells [46]. 

• Thrombin

Thrombin, or factor IIa, is a serine protease that represents one of the most important factors of the coagulation system. This serine protease converts soluble fibrinogen in insoluble strands of fibrin during clot formation and retraction. Thrombin also activates other coagulation factors, such as factors V, VIII, XI and XII, that amplify the coagulation response. Thrombin represents the most potent activator of platelets. By thrombin’s proteolytic activity, it can cleave platelet PAR receptors PAR1 and PAR4, which lead to platelet activation and aggregation. The secretion of thrombin by non-small-cell lung cancer cell lines is responsible for platelet aggregation. In contrast, TCIPA in small-cell lung cancer cell lines was fully abolished only after inhibition of ADP and thrombin activity using apyrase and hirudin [47]. 

• Thromboxane A2

TXA_2_ is a platelet agonist, and its receptor TXR is expressed on platelets. Overexpression of thromboxane receptors and thromboxane synthase (TXS) has been reported in various types of cancers including colorectal, prostate, bladder, papillary thyroid and non-small-cell lung carcinomas [48,49,50,51,52]. Thromboxane synthase catalyzes the conversion of PGH_2_ to TXA_2_. Sakaï and collaborators showed that TXS was up-regulated in the tissue of human colorectal carcinomas and that TXA_2_ increased cancer cell proliferation. The inhibition of TXS activity reduced tumor proliferation and induced apoptosis, which was rescued by addition of TXA_2_ [48]. Leval and collaborators also demonstrated that different cancer cells, including osteosarcoma cells MG-63, secreted TXA_2_ [53]. The pharmacological inhibition of TXA_2_ synthesis or of its receptor suppressed MG-63 cell TCIPA and the secretion of TXA_2_ by activated platelets. Furthermore, inhibition of TXA_2_ synthesis reduced formation of metastasis in vivo in a metastasis experimental mouse model [54]. These observations indicate a role of tumor cell-released TXA_2_ in the aggregation of platelets and in successful metastatic outbreak [53,54]. 

• CD40/CD40L

CD40, a member of Tumor Necrosis Factor (TNF) receptor superfamily, is constitutively expressed on the surface of resting and activated platelet, endothelial cells, monocytes, dendritic cells, B cells, fibroblast and renal epithelial cells [55,56]. It was originally identified in a bladder carcinoma cell line [57]. Subsequently, it was described in numerous cancer types, including ovarian, bladder, nasopharyngeal, colon, lung and breast carcinomas and in melanoma [58,59,60]. Its ligand, CD40L (also named CD154), is present in resting platelets. Following platelet activation, CD40L is quickly translocated to the membrane where it is cleaved and released in soluble form (sCD40L or sCD154) in a CD40 dependent manner [56,61]. CD40L has been shown to play several roles in inflammation and immune activation including the induction of inflammatory response in endothelial cells and the activation of immunoglobulin class switching [62]. Platelet CD40 interaction with sCD40L induces platelet activation and aggregation [63]. CD40 and CD40L are co-expressed in melanoma cells and in some carcinomas [64,65,66]. In 2002, Bussolati and collaborators demonstrated that renal carcinoma cells released sCD40L in the culture supernatant [60]. Moreover, the plasma levels of sCD40L was significantly increased in cancer patients [32,33,34,35,36]. However, the cellular origin of the measured sCD40L in the plasma of patients was not clearly identified [58], although 95% of circulating sCD40L may came from platelets [67]. 

• Cathepsin and MMPs

Matrix metalloproteinases (MMPs) are members of zinc- and calcium-dependent endopeptidases that can degrade most of the components of the extracellular matrix. These enzymes are involved in all of the steps of cancer progression from primary tumor development to metastasis in distant organs. Cancer cell MMP-2 membrane expression was observed in different cancer types, such as fibrosarcoma, colorectal and breast carcinomas. In all of these studies, authors have showed that platelet and cancer cell MMP-2 cell surface expression contribute to TCIPA [46,68,69]. Paul Jurasz et al. demonstrated that the MMP-2 dependent mechanism of the HT-1080 fibrosarcoma cell to induce TCIPA interacts with thromboxane and ADP [68]. Thus, the major pathways of platelet aggregation interact to induce TCIPA. Moreover, in this study, authors showed that nitric oxide inhibits HT-1080 cell TCIPA through the cyclic GMP dependent mechanism [68].

Cancer cells can also secrete cathepsin cysteine protease, such as cathepsin B and K. Cathepsin B levels are raised in the serum of cancer patients with different types of cancer [70]. B16 and B16a melanotic melanoma cells are known to release cathepsin B-like cysteine proteinase, and its level is positively correlated with the metastatic potential. Cathepsin B and K are both described to contribute to TCIPA in melanotic melanoma and in breast carcinoma [70,71]. 

#### 2.2.2. Tumor Cell Procoagulant Proteins

• Tissue Factor

Tissue factor (TF) is a transmembrane glycoprotein involved in the initiation of the extrinsic pathway of normal blood coagulation. TF can bind factor VIIa (TF/fVIIa) to form an active complex responsible for the proteolytic activation of factors IX and X. Once activated, factor Xa converts pro-thrombin to thrombin. This extrinsic pathway activation is required for normal blood coagulation. In normal cells, such as endothelial cells and monocytes, TF is expressed only after adequate stimulation [72]. However, TF’s overexpression is described in numerous malignant tissues and is correlated with progression to invasive cancer [73]. The expression of TF by tumor cells in the tumor microenvironment is correlated with increased levels of circulating TF antigen in cancer patients [40]. TF represents the major effector of TCIPA and is mostly responsible for the pro-thrombotic state of cancer patients [74].

• Fibrinolysis proteins

Fibrinolytic system molecules play an important role in the maintenance of hemostatic balance. In the same way as the coagulation system, cancer cells express numerous molecules involved in the fibrinolytic system. For example, urokinase type plasminogen activator (uPA) and its inhibitor PAI-1 (plasminogen activator inhibitor type 1) are both expressed in various tumor types, including prostate, colorectal, breast and ovarian carcinomas [75,76,77,78]. The increased production of fibrinolysis inhibitor PAI-1 constitutes another mechanism involved in TCIPA and in cancer-associated thrombosis [79]. Moreover, based on Cox analysis, elevated plasma levels of PAI-1 had a negative prognostic impact in terms of relapse free survival (hazard ratio: 2.5 *p*_value_ = 0.021) and overall survival (hazard ratio: 2.7 *p*_value_ = 0.002) in breast cancer patients [78].

#### 2.2.3. Microvesicles

Microvesicle is a generic term regrouping three types of vesicles: microparticles, exosomes and apoptotic bodies. Microparticles (MPs) are small membrane vesicles (0.1 to 1 µm of diameter) released from eukaryotic cells upon stimulation, apoptosis or oncogenic transformation. These particles are composed of anionic phospholipids, including phosphatidylserine and phosphatidylethanolamine, that represent an adequate surface to initiate and support the coagulation and the fibrinolytic system. The particles also express cellular origins antigens, cytokines, matrix metalloproteinases and content mRNA and miRNA. Plasma MPs in healthy patients are generally derived from circulating blood cells, such as platelets, red blood cells, leukocytes and endothelial cells. Platelet MPs (PMPs) represent 70% to 90% of plasma-born MPs [80]. 

Chargaff et al. described a precipitable factor for the first time in 1949, which were PMPs that could accelerate thrombin generation in platelet-free plasma [81]. Today, it is well-established that TF positive tumor-derived MPs are associated with the pro-thrombotic state of cancer patients [82,83,84]. Numerous studies reported an increase of circulating MPs levels, including platelet, endothelial cell and tumor-derived MPs in different types of cancer, including gastric, pancreatic, colorectal, lung, ovarian and breast cancers and an increased procoagulant activity [84,85,86,87,88,89]. Cancer cell-derived MPs, as well as origin cells, can express all of the molecules involved in platelet activation and aggregation and in the activation of the coagulation cascade. By the exposure of phosphatidylserine and the expression of procoagulant proteins, such as TF, cancer cell-derived MPs contribute to intravascular thrombin generation. Hron et al. demonstrated a two-fold increase of TF bearing MPs in colorectal patients in comparison to control. The level of TF bearing MPs were positively correlated with the D-dimer level as a marker of clotting activation [84]. More recently, our team described a specific signature of MPs in colorectal and pancreatic cancer patients compared to inflammatory bowel or pancreatic diseases and healthy patients. The microparticulosome signature showed a significant increase of plasma fibrin positive MPs in pancreatic cancer patients in comparison with the other groups [90]. These results were in accordance with the higher risk of thrombosis in this type of cancer. Moreover, we showed in a mouse arterial thrombosis model that pancreatic cancer cell-derived MPs bearing PSGL-1 (P-selectin ligand 1) and TF accumulate to the site of injury and accelerate thrombus formation in vivo. In addition, in different mouse model of venous thrombosis, TF positive tumor cell-derived MPs were described as effectors of platelet activation and thrombus formation in a thrombin-dependent manner [91,92]. These MPs were able to interact with neutrophil extracellular traps (NETs) that are known to play several roles in cancer-associated thrombosis [93]. TF positive cancer cell-derived MPs are also able to transfer TF to endothelial cells, increasing the procoagulant potential of endothelial cells [94]. Thus, TF cancer cell derived MPs seem to actively participate in thrombotic events in cancer patients. 

Exosomes are smaller vesicles than microparticles with a diameter of 40 nm to 100 nm. Exosomes differ from microparticles by their size, formation process and protein expression. Recently, it has been shown than exosomes derived from 4T1 breast cancer cells were able to induce NET formation on neutrophils from G-CSF treated mice. In different mouse model of arterial and venous thrombosis, a blood infusion of 4T1 tumor derived exosomes in G-CSF treated mice reduced the occlusion time and accelerated thrombus formation in vivo [95]. Thus, tumor-derived exosomes and neutrophils can cooperate in vivo to contribute to cancer-associated thrombosis. 

#### 2.2.4. Adhesive Proteins

Cancer cells express many adhesive molecules that enable their interaction with the blood host cells, including platelets, endothelial cells and immune cells. To date, there are few mechanisms described for the interaction of cancer cells with platelets in the bloodstream.

In 1988, Karpatkin et al. showed the existence of platelet integrin α_IIb_ß3 inhibition by blocking antibody reduced colorectal and melanoma cancer cell–platelet interactions in vitro and reduced metastasis in vivo. A fibronectin and Von Willebrand factor blockade by RGDS (Arg-Gly-Asp-Ser peptide) also decreased the interactions between platelets and cancer cells, suggesting an interaction via these adhesive proteins in an α_IIb_ß3-dependent manner [41]. Moreover, it has been shown that tumor cell integrin αvß3 can bind platelet integrin α_IIb_ß3 and mediates cancer cell-platelet interaction and aggregation [96,97]. More recently, Mammadova-Bach et al. showed that platelet integrin α6ß1 directly interacts with colorectal MC-38 and breast cancer AT3 cell ADAM9. Using an orthotopic cancer model and metastasis experimental model, they demonstrated that these interactions actively control lung metastasis but not tumor growth. Platelet integrin α6ß1–tumor cell ADAM9-dependent interaction leads to platelet activation, granule secretion and extravasation of cancer cells in the lungs [98].

P-selectin (CD62P) is a member of Ca^2+^-dependent adhesion molecules belonging to the family of the animal lectins (C-type lectins). In general, selectins are adhesion molecules that mediate intercellular interactions among leukocytes, platelets, cancer cells and vascular endothelial cells. P-selectin is stored in alpha granules of platelets and in Weibel Palade bodies of endothelial cells. Following cellular activation and degranulation, P-selectin is rapidly expressed on the membrane of platelets and endothelial cells. P-selectin interacts with mucinous ligand PSGL-1 expressed mostly on leukocytes and cancer cells. P-selectin can also bind a small glycoprotein, known as sialyl-Lewis X (s-Le X), mostly present on mucinous cancer cells. Using P-selectin knock-out mice, Kim et al. showed that platelets can interact with cancer cells in a P-selectin-dependent manner [99]. P-selectin deficiency reduced tumor growth and lung homing metastasis in vivo. This effect was due to an abolition of platelets infiltrating into solid tumors, a decrease of angiogenic factor secretion, such as VEGF, and a decrease in the number of platelets-cancer cells aggregates in the bloodstream [99,100]. Moreover, it has been shown that P-selectin dependent aggregation of platelets around tumor cells led to the transfer of procoagulant MPs between cells that subsequently contributes to fibrin deposition and microthrombi formation [101]. In addition, up-regulation of P-selectin in cancer patients was identified in 1991 and was proposed as a biomarker for cancer associated thrombosis [32,102].

Glycoproteins (GPs), expressed on both platelets and cancer cells, are known to mediate cancer cell–platelet interactions. The GPIbα, which is a component of the platelet receptor GPIb-V-IX, was reported to contribute to TCIPA and tumor progression, but its specific role remains contradictive. On one hand, the inhibition of GPIbα by blocking antibodies partially blocks TCIPA in a dose dependent manner [103,104]. Moreover, mice that lack functional GPIb-IX receptors showed a 15-fold reduction of lung metastasis using B16F10.1 melanoma cells in comparison to control mice [105]. Furthermore, MCF-7 breast cancer cell GPIbα expression and its role in TCIPA was also reported [106]. On the other hand, Karpatkin et al. showed that inhibition of GPIbα by blocking antibodies has no effect on colorectal cancer cell induced platelet aggregation [41]. In addition, in vivo antibody mediated GPIbα inhibition increases the lung metastasis of melanoma cells, which was abolished in P-selectin deficient mice [107]. Thus, the contribution of GPIbα in the interaction of platelets with cancer cells and in TCIPA remains unclear and should be verified in the future.

Podoplanin (PDPN) is a mucin-type sialoglycoprotein. PDPN, which was initially described in the lymphatic vessel formation during embryogenesis, is upregulated in various types of cancer, including colorectal, bladder and lung carcinomas and contributes to TCIPA, tumor growth and metastasis. Podoplanin can directly bind the platelet receptor C-type lectin-like receptor (CLEC-2) and induces platelet activation and aggregation [108,109,110,111,112]. 

#### 2.2.5. Involvement of Activated Platelets in Cancer-Associated Thrombosis

As discussed below, tumor cells can activate platelets and the coagulation system by direct interaction in the bloodstream or indirectly via microvesicles and/or secreted factors. Once activated, platelets display a procoagulant surface by the expression of anionic phospholipids participating in thrombin generation, fibrin formation and clotting [113]. The generation of Platelet-rich microthrombi in adenocarcinoma bearing mice appeared to be dependent on platelet-leukocyte interactions since the formation of microthrombi was diminished in P- or L-selectin deficient mice [114]. Furthermore, platelets-neutrophils interactions were required for NETs formation [115]. In a mouse model of transfusion-related acute lung injury (TRALI), the inhibition of platelet activation by both aspirin and tirofiban significantly decreased NETs formation, indicating that platelet activation is required for NETs formation [116]. Using P-selectin deficient mice, Etulain et al. recently showed that, platelet P-selectin–neutrophil P-selectin Ligand 1 (PSGL-1) interactions were necessary for the induction of NETs [117]. Subsequently, in different animal models, it was demonstrated that platelet-induced NETs formation also involved the adhesive proteins GP1b and the ß2-integrin [118,119]. Alternatively, platelets could also secrete inflammatory molecules, such as HMGB1 (High-Mobility Group Box 1), ß-defensin and CXCL4 (CXC chemokine ligand 4) involved in the formation of NETs in vitro and in vivo [118,120,121,122]. 

Using CD40 melanoma positive cells, Amirkhosravi and collaborators demonstrated that activated platelet supernatants containing sCD40L, recombinant soluble CD40L, as well as activated platelets-tumor cells interactions increased procoagulant activity and TF antigen expression in tumor cells and in monocytes [123]. The increased procoagulant TF activity was inhibited by anti-CD40L antibody, indicating that the CD40-CD40L interaction may possibly enhance intravascular coagulation and hematogenous metastasis [123]. Altogether these findings indicate a cross-talk between inflammation and platelet-dependent thrombosis in cancer, confirming the emerging concept of immunothrombosis. Thus, activated platelets, by providing pro-coagulant surface, by releasing inflammatory molecules, by interacting with neutrophils and by inducing NETosis, actively participate to cancer- associated thrombosis. 

### 2.3. Tumor Educated Platelets

The notion of tumor-educated platelets (TEPs) is an emerging concept that provides interesting tools for cancer diagnostics. Indeed, platelet RNA profile changes were reported in several cancer types, including lung, prostate, glioma and breast carcinomas [124,125,126]. However, the molecular mechanisms involved in the RNA and protein profiles modifications during cancer progression have not yet been well elucidated.

#### 2.3.1. Platelet RNA Profiles in Cancer Patients

Platelets contain a rich repertoire of RNA species, including mRNA (premRNA and mRNA), ribosomal RNA (rRNA), microRNA (miRNAs), small nuclear RNA (snRNA), small nucleolar RNA (snoRNA), antisense RNAs (asRNA) and transfer RNA (tRNA) [127,128,129,130,131,132,133]; the ability of platelets to translate mRNA into proteins has long been known [134,135]. Indeed, platelets possess a functional spliceosome and spliceosome factors that process the premRNAs within the nuclei of other cell types. External stimuli, such as activation of platelet receptors, induce the specific splicing of premRNAs in circulating platelets. For example, Denis et al. showed that the activation of platelets by thrombin in the presence of a fibrinogen matrix induced specific splicing and translation of Il1-ß premRNA [127]. In LPS-stimulated platelets, the splicing initiation of Il-1ß premRNA appears to be dependent on Jun and Akt phosphorylation [136]. The stimulation of platelets with bacteria such as staphylococcal alpha-toxin or LPS was also shown to induce the splicing of platelet TF-premRNA in a Cdc2-like kinase phosphorylation-dependent manner [137]. In nucleated cells, snoRNAs accumulate in the nucleolus and are implicated in translation and splicing by their abilities to modify the nucleotides of snRNA and rRNA molecules [138]. In nucleated cells, the snoRNA SNORD-115 was recently described to influence the alternative splicing of the serotonin receptor 2C premRNA [139]. As indicated below, platelets contain snoRNAs and splice factors which may indicate that platelet snoRNAs mediate alternative splicing [132]. Thus, the activation of platelets by external signals induces specific splice variants of premRNA into platelets, providing platelets with a specific mRNA signature that enables potential applications in cancer diagnostics. However, the direct effects of tumor cells and/or tumor microenvironment signals on the specific splicing and translation of platelet premRNAs remains unknown and must be investigated. 

In the literature, the main way in which primary tumors influence platelet RNA profiles appears to be through the uptake of tumor-derived microvesicles. For example, Calverey et al. reported in 2010 that platelet gene expression was significantly downregulated in metastatic lung cancer [124]. Indeed, microarray analysis of platelet RNA profiles from healthy individuals and metastatic lung cancer patients have revealed 200 affected RNAs, with 197 downregulated RNAs in cancer patients, and the authors proposed that platelet RNA profiles may be useful for the detection of metastatic lung cancer. Subsequently, Nilsson et al. demonstrated that the in vitro platelet uptake of U87/U87-EGFRvIII glioma and 22RvI prostate cancer-derived microvesicles contained mutant RNA [84]. Moreover, these researchers showed that blood platelets isolated from glioma and prostate cancer patients contain tumor-derived RNA biomarkers EGFRvIII and PCA3, respectively. Interestingly, the researchers also revealed a distinct RNA signature in platelets from glioma patients compared to healthy individuals, which may be attractive for glioma detection [125]. Furthermore, this team recently showed by RT-PCR the presence of EML4-ALK (Echinoderm Microtubule-associated protein-Like 4—Anaplastic Lymphoma Kinase) RNA rearrangement in blood platelets from non-small-cell lung cancer (NSCLC) patients with 100% specificity and 65% sensitivity. In this study, RNA detection by RT-PCR of plasma EML4-ALK rearrangements exhibited a lower sensitivity than platelets (21%), which demonstrates the usefulness of platelets in the detection of cancer biomarkers [140]. The team also demonstrated by confocal microscopy and RT-PCR that tumor-derived exosomes are able to transfer EML4-ALK rearranged RNA into platelets in vitro. EML4-ALK rearrangements in platelets were correlated with progression-free and overall survival in 29 crizotinib treated patients. Among the 29 crizotinib treated patients, the progression-free survival was 3.7 months and 16 months for patients with EML4-ALK positive platelets and EML4-ALK negative platelets, respectively. Moreover, the authors demonstrated, in the platelets of one representative patient, that the monitoring of EML4-ALK rearrangements over a period of 30 months may predict crizotinib outcomes two months prior to radiographic disease progression [140].

Best et al. using RNA-seq analysis, termed ThromboSeq technique, evaluated TEPs mRNA profiles in 228 patients with localized and metastasized tumors in comparison to 55 healthy individuals. After the selection of spliced RNA reads and the exclusion of genes with low coverage, 5003 different protein coding and non-coding RNAs in platelets of healthy donors and cancer patients were detected. Among the 5003 RNAs, 1,453 were increased and 793 were decreased in TEPs compared to healthy donor platelet samples. DAVID and CAGE gene ontology algorithms revealed a downregulation of RNAs involved in RNA metabolism and RNA splicing in TEPs. The gene ontologies also correlated to platelet activation, platelet vesicle transport, ATP signaling and cytoskeleton activation, all of which potentially reveal the activation status of platelets during cancer progression.

In this study, the authors were able to distinguish cancer patients from healthy individuals with 96% accuracy. Moreover, they correctly identified the location of tumors across six tumor types (NSCLC, glioblastoma, colorectal carcinoma, breast cancer, hepatobiliary cancer and pancreatic cancer) with 71% accuracy. Furthermore, using surrogate TEP mRNA profiles, mutant oncogenic drivers including KRAS, EGFR, PIK3C4, MET and HER-2 positive tumors were precisely distinguished, demonstrating that platelets can also assist in determining the tumor type and molecular subclass and in selecting patients for targeted therapies [126]. Recently, Best et al. demonstrated that particle-swarm optimization algorithms enable efficient selection of RNA biomarker panels to diagnose early and late-stage NSCLC from TEPs independently of age, smoking habits, whole-blood storage time and various inflammatory conditions [141]. 

In addition to mRNA, platelets also contain non-coding RNAs and small RNAs. Best and colleagues also analyzed the differentially expressed non-coding RNAs and revealed 20 altered genes that showed a tumor-specific profile. Among the 20 differentially expressed non-coding RNAs, 16 were upregulated in TEPs. Interestingly, two of the downregulated non-coding RNA are associated with proliferation of tumor cells. For example, one of the downregulated RNAs is the growth arrest specific transcript 5 (GAS5), involved in cell proliferation, and its downregulation has been shown to be pro-cancerous in several tumor types [142,143]. Recently, Luo et al. evaluated the differential expression of LncRNA, including MAGI2 antisense RNA (MAGI2-AS3) and ZNFX1 antisense RNA1 (ZFAS1), in platelets from healthy individuals and NSCLC patients. Their results showed that MAGI2-AS3 and ZFAS1 were significantly downregulated in platelets from NSCLC patients compared to healthy controls. In this study, the clinicopathologic characteristics analysis revealed that the level of MAGI2-AS3 correlated with tumor-node-metastasis stage (TNM), lymph-node metastasis and distant metastasis, while ZFA1 level was correlated only with TNM [144].

Increasing evidence has demonstrated the roles of microRNA in cancer biology. In 2012, Plé et al. described 532 different microRNAs in platelets [133]. MicroRNA also play important roles in platelet function. For example, the miR-126 has been shown to directly and indirectly affect ADAM-9 and P2Y12 expression. Its inhibition in mice significantly reduced platelet aggregation, suggesting that micro-RNAs are linked to platelet reactivity [145]. Moreover, in cardiovascular disease, platelets from myocardial infarction patients differentially expressed nine microRNAs compared to healthy controls [146]. These observations suggest that microRNA signatures in platelets could be useful as diagnostic tools in cardiovascular diseases and cancer.

Thus, the blood-based “liquid biopsies” and the thromboSeq techniques constitute effective tools for pan-cancer, multiclass cancer and companion diagnosis and for the monitoring of treatment response. However, microvesicle independent mechanisms of RNA transfer, miRNA transfer and their role in platelet biology and cancer remain to be investigated. 

#### 2.3.2. Platelet Protein Profiles in Cancer Patients

As described below, platelets exhibit the ability to translate mature, spliced RNA into proteins [147,148,149]. During the previous decades, numerous studies investigated the extensive protein profiles of platelets. However, Londin et al. showed that no corresponding protein was identified for almost 60% of platelet detected mRNA [147,150]. This suggests that not all mRNAs are translated in proteins in platelets. Moreover, there is no study showing the translation of tumor-derived mRNA transferred into platelets. To date, studies only elucidated the transfer of tumor-derived RNA into platelets and their potential applicability to cancer diagnostics, but no associations with the protein profile of platelets were examined [124,125,140]. Only a few studies demonstrated that platelets, by their abilities to ingest biomolecules, express or sequester protein from the primary tumors. 

The most described mechanism of platelet protein uptake and storage is through the endocytic pathway. Indeed, endocytosis is the key pathway to fibrinogen uptake and the trafficking of integrins and purinergic receptors into platelets. In 2012, Hung and collaborators reported that fibrinogen uptake is associated with the expression, mediated through a clathrin-dependent manner, of integrin αIIbß3 and of the endocytic adaptor protein Disabled-2 (DAB2). The knockdown of DAB2 as well as clathrin inhibitor chlorpromazine significantly reduced the uptake of fibrinogen [151]. More recently, the cellubrevin/vesicle-associated membrane protein-3 (VAMP-3) was identified as an element of the platelet endocytic machinery involved in fibrinogen uptake. In vitro and in vivo experiments showed that loss of VAMP-3 led to a robust defect in the uptake and storage of fibrinogen into platelets. Moreover, VAMP-3 null mice display lower levels of platelet-associated fibrinogen than control mice [152]. It is possible that platelets utilize endocytic machinery to ingest tumor-derived biomolecules; however, further studies are required to understand the exact mechanisms.

Klement et al. demonstrated that platelets actively sequester angiogenesis regulators, such as VEGF and bFGF, in vitro and in vivo in cancer bearing hosts. In vitro, the incubation of platelets with VEGF, bFGF and endostatin revealed that platelets accumulate these proteins into their cytoplasmic and granular compartments. Using subcutaneous Matrigel pellets containing ^125^I-labeled VEGF, it was confirmed that platelets accumulated ^125^I-VEGF at higher concentrations than those found in plasma or various organs. Moreover, they showed that tumor bearing mice possessed an elevation of VEGF levels in platelets compared to control mice [153]. Furthermore, this higher level of VEGF into cancer patient platelets compared to healthy control was also observed in breast cancer by Holmes and collaborators. They also showed that breast cancer and its treatment induce platelet phenotype changes. Indeed, agonist induced release of VEGF was greater in cancer patients compared to healthy controls despite a decrease in the efficiency of VEGF secretion in patients with cancer (21% of the total VEGF platelet pool for cancer patients using ADP stimulation versus 37% for healthy controls). Intriguingly, in patients with breast cancer, platelet inhibition by cangrelor was greater in patients with breast cancer compared to healthy controls, suggesting that lower doses of cangrelor could be used in patients to avoid bleeding risks [28]. 

Another example was recently showed in the field of cancer immunotherapy. Regarding the large fraction of patients who do not respond to checkpoint inhibitors, some authors have focused on platelet and peripheral blood mononuclear cell (PBMC) programmed death-ligand 1 expression (PD-L1). Interestingly, these authors showed that platelets and PBMC from smokers and patients with lung cancer and head and neck squamous cell carcinoma (HNSCC) express and upregulate PD-L1 independently of tumor stage. In four patients with lung cancer, treatment with a fully humanized monoclonal antibody against PD-L1, atezolizumab, induced a decrease of PD-L1 platelet expression which was restored over 20 days. These findings could partially explain why some patients respond poorly, or not at all, to checkpoint immunotherapies; the results also strongly suggest that PD-L1 platelet expression could be used as a biomarker to predict successful therapeutic outcome [154].

### 2.4. Antiplatelet and Platelet Based Therapies

#### 2.4.1. Antiplatelet Therapies

The role of platelet activation in cancer-associated immuno-thrombosis and in successful metastatic outbreak suggests that TCIPA represents a potential therapeutic target in the management of CAT and tumor progression. Over the last few decades, numerous clinical studies suggested that taking daily aspirin reduced cancer incidence, metastasis and mortality, especially for colorectal cancer [155]. However, these findings were discussed and debated during the last century. The acetylsalicylic acid, which correspond to the active substance of aspirin, inhibits COX enzymes involved in the generation of TXA2. Aspirin is commonly used in clinic to treat patients with coagulopathies. Recently, using an experimental metastasis mouse model in immunodeficient mice, Guillem-Lobat P and collaborators demonstrated that low-dose aspirin reduced lung metastatic seeding by averting the enhanced proaggregatory effects induced by platelet-tumor cells interactions [156]. In 2016, two large US prospective studies performed on more than 130,000 patients with a follow-up time of 30 years showed that regular use of low-dose aspirin (two times per week) was associated with a lowered risk for overall cancer incidence, and especially for gastrointestinal tracts cancers [157]. These observations suggest that low-dose aspirin could be used to prevent cancer development with limited risks of bleeding or other side effects. Numerous other studies are currently in progress to evaluate the potential role of aspirin in cancer treatment [158]. However, the exact mechanisms by which aspirin exerts antitumor effects have to be investigated. Another way to inhibit platelet activation in cancer patients is to target the ADP-receptors on platelets with P2Y12 inhibitors such as clopidogrel or ticagrelor. Some studies performed on cancer mouse models have demonstrated that the use P2Y12 inhibitors, such as ticagrelor or clopidogrel, reduced tumor growth and metastasis in vivo [159,160]. The use of clopidogrel in combination with chemotherapeutic drugs enhanced vascular permeability and drug delivery [161]. Other studies, however, showed no effect of clopidogrel alone on the tumor growth of three cancer types, including breast, colorectal and prostate cancer, or, even worse, a pro-tumoral effect of prasugrel [161,162]. Altogether the results observed may depend on the model studied and the design of the study. Recently, a large retrospective study performed on more than 180,000 patients revealed that the use of clopidogrel with or without aspirin was associated with lower incidence of cancer [163,164]. Additional clinical studies are needed to determine the anti-cancer potential of P2Y12 inhibitors.

#### 2.4.2. Platelet-Based Therapies

Platelets and platelets released factors play several roles in cancer progression including immune tumor evasion, tumor growth and metastasis [164]. In numerous inflammatory pathologies, including atherosclerosis and graft reject, platelets seem to actively participate to the inflammatory and immune response [165,166,167,168]. Moreover, it was recently showed that platelets extravasated in tumor microenvironment and were associated with the tumor vessel structure [19,42,100,169]. Based on these observations, some experimental studies investigated the abilities of platelets to deliver potential anti-tumoral signals into the microenvironment or in the bloodstream during their interaction with circulating cancer cells. Li and col. recently showed that intravenous infusion of IFN-γ (Interferon-γ) induced protein 10 (IP-10) rich platelets in a mouse model of melanoma significantly reduced tumor growth in vivo. They demonstrated that platelets directly delivered IP-10 in the microenvironment of the tumors and suppressed the recruitment of the immunosuppressive FoxP3 positives T cells [170]. The genetic engineering of platelet which were able to express the tumor necrosis factor related apoptosis-inducing ligand (TRAIL), was shown to induce the apoptosis of cancer cells in vitro and significantly reduced metastasis in vivo [171]. In front of these promising experimental results, the potential of platelets to deliver chemotherapeutic drugs or anti-tumoral signals directly in the microenvironment should be deeper investigate in the future.

## 3. Conclusions

**Figure 2:**
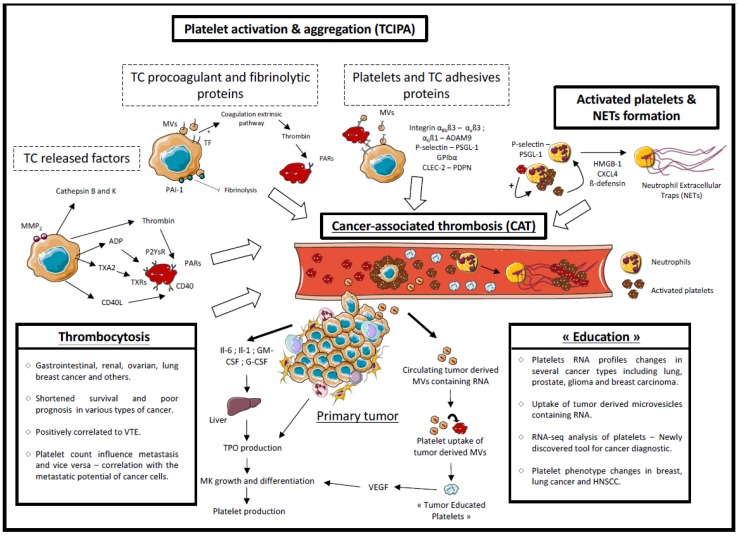
Impacts of cancer on platelet production, activation and education and mechanisms of cancer-associated thrombosis. This figure summarizes all the mechanisms involved in the production, the activation and the education of platelets mediated by the primary tumor and the mechanisms of cancer-associated thrombosis. TC: Tumor Cell. This figure was obtained using Servier medical art. http://smart.servier.com/.

Finally, in response to all of the biomolecules released and/or expressed by tumor cells and tumor microenvironment, the notion of tumor-educated platelets is particularly appropriate. Cancer cells can mediate a paracrine activity to increase platelet production that is not harmless. In addition, through oncogenic transformation, cancer cells have the ability to constantly produce MPs, which are released in the bloodstream and participate in (i) platelet activation and RNA profile changes and (ii) contribute to pro-thrombotic states in cancer patients. Thus, directly and indirectly, cancer cells can modify the physiology and the phenotype of platelets that is closely associated with the pro-thrombotic state of cancer patients (summarized in Figure 2). Furthermore, the mechanisms of TCIPA provide numerous advantages to the primary tumor and the circulating cancer cells. Indeed, platelet activation leads to the release of active biomolecules content on their granules, all of which participate in tumor progression and successful metastatic outbreak. As discussed in this review, platelet RNA profile changes constitute a new and promising area of cancer diagnostics even if larger cohort studies are necessary for the future clinical utilization of TEPs. However, the functionality in cancer biology of platelet RNA profile changes is still unknown and must be investigated. The expression of clinically targetable oncogenic proteins by platelets should be further investigated in the future in order to predict therapeutic outcomes.
